# Provider Perspective on Being Recorded During Emergency Medicine Discharge Conversations

**DOI:** 10.7759/cureus.24523

**Published:** 2022-04-27

**Authors:** Nickolas Meier, Andrew Little, Teresita Morales-Yurik, Brandon Arehart

**Affiliations:** 1 Emergency Medicine, OhioHealth Doctors Hospital, Columbus, USA; 2 Emergency Medicine, Adventhealth East Orlando, Orlando, USA

**Keywords:** patient-oriented, instructions, recording, discharge to home, ed discharge

## Abstract

Objective: Despite the possible benefits of provider-recorded visit summaries for patient use, the utilization of such recordings has yet to be adopted as standard care in most specialities. The objective of this study was to investigate the perspectives of emergency department (ED) providers regarding the utilization of audio or video recordings during ED patient encounters, particularly during discharge conversations.

Methods: This study utilized an eight-question survey pooling the opinions of various emergency medicine nurses, advanced practitioners, residents, and physicians within a local Ohio hospital system. Providers from multiple healthcare centers were studied. Study data were collected using an anonymous online survey database.

Results: Fifty-seven providers were surveyed. Twenty (35%) agreed that patients had the right to record medical conversations, and 36 (63%) cited potential legal liability as their reason for hesitation. Twenty-five providers (43.9%) answered that no video or audio recordings should be provided at discharge. There was no significant difference in secondary outcomes comparing between demographic categories of age, sex, and practice facility type (p>0.05).

Conclusion: Providers who responded to this survey did not feel comfortable being recorded during any portion of their patient encounter, even when providing discharge instructions. Our study showed that there is at least some hesitation on behalf of ED providers based on fear of legal retaliation or violation of HIPPA. However, more research in this area is necessary for recordings to become the standard of care during ED encounters.

## Introduction

In 1977, Dr. Hugh Butt proposed using audio-recorded discharge instructions to strengthen doctor-patient communication [1]. Patients at the time were reporting dissatisfaction and misunderstanding of many medical conversations between providers and patients [1]. Since then, technological advancements have made electronic recordings more common in healthcare, but only in niche fields. The use of video recordings has steadily increased in the operating room where the ability to replay procedures serves to document steps, educate, and ensure quality control [2,3]. Likewise, audio recordings became more popular in emotionally-charged environments, like oncology, as the tape allows the patient to review the information at a later time [3-7].

Outside of operating rooms and oncology clinics, the use of video and audio recordings waned in the mid-1990s [2,3,6]. Campbell et al. reported a decrease in video recordings in trauma centers from 58% to 18% [6]. The Health Insurance Portability and Accountability Act (HIPAA) of 1996 has been cited as the primary reason for the abandonment of recording procedures in some trauma rooms and outpatient offices for fear of jeopardizing patient confidentiality [3,6]. Ellis et al. also cite issues with acquiring adequate personnel and a lack of staff support as reasons for the decline in provider-initiated recording practices during trauma resuscitations [2]. This study noted that the lack of staff support stemmed from their fear of repercussions and being singled out for poor performance [2]. Some other studies have demonstrated a lack of staff support and patient-privacy laws as the main inhibitors to incorporating this technology [2-4,6-13]. 

In addition to privacy concerns and a lack of staff support, McConnell et al. note that most surgeons and general practitioners are opposed to providing patients with an audiotape recording of their consultation, stating that it is medicolegally “risky” for fear of legal repercussions regarding the patient encounter [4]. Other authors share this concern [10-12]. Lastly, some providers also believe operating a recording device would lengthen the patient encounter and perhaps make patients present medical information not as openly [4,8]. 

Despite the possible benefits of provider-recorded summaries for patient use, their incorporation has stagnated outside of a select few fields. In the ED, the high stress and fast-paced environment mean patients may benefit from a recorded discharge summary for reasons mentioned prior. The American College of Emergency Physicians (ACEP) acknowledges the potential benefits and even encourages its incorporation in EDs nationwide [14]. However, there is sparse literature on this topic specific to the ED environment. Though it may be worth considering the incorporation of recorded instructions into the ED patient discharge process, it is first necessary to better understand the perspectives of ED providers concerning this process change.

Discharge instructions serve as an essential step in the ongoing management of patients released from the emergency department. They are meant to help patients understand their medical course, and provide them with treatment plans, medication regimens, follow-up guidance, and reasons for returning to the ED. To maximize patient care outcomes, this information needs to be effectively communicated from the practitioner to the patient and/or family. Options to record components of the ED encounter, including the discharge conversation, may help patients better retain this information and improve health outcomes. 

The patient-perceived benefits of these recordings have been reported in some studies. Patients self-report that they remember more, feel more at ease with their medical condition, are overall more satisfied with their medical consultation, and value the opportunity to share their recordings with family and friends [1,4,5,7-9]. When comparing audio recordings to a summary letter, patients preferred the former [4,7]. The rationale is that, compared to the letter, an audiotape is “more personal, reassuring, and human” [4,7]. Conversely, 71% of oncologists, surgeons, and general practitioners felt an individualized letter would, rather than an audio recording, be more effective at summarizing the visit with the patient [2,4]. The literature on ED clinician perspectives on recording patient encounters is limited.

The purpose of this study was to investigate the perspective of local ED providers on patient-recorded discharge instructions. We hypothesized that the majority of ED providers would prefer not to be recorded during these patient interactions, and would likely cite medicolegal reasons for their hesitation.

## Materials and methods

This study, reviewed and approved by the OhioHealth Institutional Review Board (IRB), utilized an eight-question online survey which was circulated to several EDs within the same hospital system around the central Ohio area. The hospitals that were studied included large tertiary care hospitals, smaller community-based hospitals, freestanding EDs, and an academic hospital. The survey was distributed to these EDs via variations of posting flyers in the ED break rooms, as well as emails sent by ED leadership to its employees. The notifications included a quick response (QR) code that provided a survey link to an online anonymous survey collected via REDCap data collection software (University of Colorado, USA). No identifying information was collected.

The study was designed to survey the opinions of emergency medicine providers, including nurses, advanced practitioners, residents, and physicians. A goal of 75 survey participants was decided based on an estimate of the expected participation rate among the EDs we surveyed. Inclusion criteria included age ≥ 18 years, English speaking and reading, and participants needed to be emergency medical providers such as Bachelor of Science in Nursing (BSN), registered nurse (RN), nurse practitioner (NP), physician assistant (PA), Doctor of Osteopathic Medicine (DO), or Doctor of Medicine (MD) for the local hospital organization. The participants that did not meet the inclusion criteria were excluded. Questions and answers from incomplete surveys were still utilized in the data set. The type of primary practice facility was chosen by the participant based on what type of environment they believe they practice in when provided with a list of predetermined choices. 

Survey responses were collected from September 1, 2020, to December 1, 2020. The data of responses was automatically generated via the REDCap data collection software. The study’s primary outcome was to establish an overall consensus of thoughts on being recorded during patient encounters, particularly during discharge conversations. As this was an initial survey of opinions, secondary outcomes included a comparison of opinions between age, sex, and primary practice setting. 

Primary outcome analysis was performed by comparing the gross response to questions in the survey based on the percentages of the survey population. Secondary outcome analyses were compared using a Kruskal-Wallis one-way analysis of variance (ANOVA), a statistical variance test. Statistical significance was determined based on a standard p-value of less than 0.05. 

## Results

A total of 57 participants were surveyed and a summary of all results of the questionnaire is presented in Table 1. Despite having a goal of at least 75 surveys collected, data collection was stopped after three months of attempting to promote voluntary participation. All 57 participants included in the study met inclusion criteria. Approximately 78% of participants were between the ages of 26 to 50. There was a reasonably equal distribution of male and female representation (30 vs. 26 respectively). Most of the participants self-identified as working in either an academic community hospital or multiple ED settings, versus the minority who answered as working in a community non-academic setting (Table 2). 

**Table 1:** Summary statistics Summary of all responses to our survey. BSN: Bachelor of Science in Nursing, RN: Registered nurse, NP: Nurse practitioner, PA: Physician assistant, DO: Doctor of Osteopathic Medicine, MD: Doctor of Medicine, HIPAA: Health Insurance Portability and Accountability Act

Characteristic	Results
Are you 18 years of age or older?	
Yes	57 (100.0 %)
Are you an Emergency Medicine Provider (BSN, RN, NP, PA, DO, or MD)?	
Yes	57 (100.0 %)
Are you employed at an OhioHealth network hospital or care center?	
Yes	57 (100.0 %)
Have you already completed this survey?	
No	57 (100.0 %)
What type of OhioHealth facility do you primarily work in?	
Academic community setting hospital	31 (54.4 %)
Multiple	20 (35.1 %)
Non-academic community setting hospital	5 (8.8 %)
Missing Values	1 (1.8 %)
Age	
18-25 years old	3 (5.3 %)
26-35 years old	24 (42.1 %)
36-50 years old	21 (36.8 %)
51-70 years old	8 (14.0 %)
Missing Values	1 (1.8 %)
Sex	
Female	26 (45.6 %)
Male	30 (52.6 %)
Missing Values	1 (1.8 %)
What is your comfort level with having discharge conversations with patients video recorded during an emergency department encounter?	
N	51
Mean±SD	43.84 ± 34.40
Range	0.00 to 98.00
Median	50.00
What is your comfort level with having discharge conversations with patient audio recorded during an emergency department encounter?	
N	47
Mean±SD	52.96 ± 33.62
Range	0.00 to 100.00
Median	64.00
To your knowledge have you ever been recorded (video or audio) by a patient during an emergency department encounter?	
No	30 (52.6 %)
Yes	26 (45.6 %)
Missing Values	1 (1.8 %)
If yes has the recording ever been used against you in a medical legal context?	
No	23 (40.4 %)
Unsure	3 (5.3 %)
Missing Values	31 (54.4 %)
Patients have the right to record medical conversations	
Agree	5 (8.8 %)
Disagree	14 (24.6 %)
Neutral	9 (15.8 %)
Slightly agree	15 (26.3 %)
Slightly disagree	13 (22.8 %)
Missing Values	1 (1.8 %)
Recordings take too much time to do	
Agree	6 (10.5 %)
Disagree	6 (10.5 %)
Neutral	20 (35.1 %)
Slightly agree	11 (19.3 %)
Slightly disagree	13 (22.8 %)
Missing Values	1 (1.8 %)
Recordings increase patient adherence to medical advice	
Agree	4 (7.0 %)
Disagree	5 (8.8 %)
Neutral	20 (35.1 %)
Slightly agree	18 (31.6 %)
Slightly disagree	9 (15.8 %)
Missing Values	1 (1.8 %)
Recordings increase the probability that patients will go to follow-up appointments	
Agree	1 (1.8 %)
Disagree	11 (19.3 %)
Neutral	19 (33.3 %)
Slightly agree	15 (26.3 %)
Slightly disagree	10 (17.5 %)
Missing Values	1 (1.8 %)
Recordings present legal liability risks that outweigh their benefits	
Agree	16 (28.1 %)
Disagree	4 (7.0 %)
Neutral	9 (15.8 %)
Slightly agree	20 (35.1 %)
Slightly disagree	7 (12.3 %)
Missing Values	1 (1.8 %)
Recordings can easily result in HIPAA violations	
Agree	22 (38.6 %)
Disagree	1 (1.8 %)
Neutral	9 (15.8 %)
Slightly agree	18 (31.6 %)
Slightly disagree	5 (8.8 %)
Missing Values	2 (3.5 %)
What part of a patient encounter should be recorded-None	
Checked	30 (52.6 %)
Unchecked	27 (47.4 %)
What part of a patient encounter should be recorded-Entire encounter	
Checked	2 (3.5 %)
Unchecked	55 (96.5 %)
What part of a patient encounter should be recorded-Discharge only	
Checked	24 (42.1 %)
Unchecked	33 (57.9 %)
What part of a patient encounter should be recorded-History and physical exam	
Unchecked	57 (100.0 %)
What part of a patient encounter should be recorded-Other	
Checked	1 (1.8 %)
Unchecked	56 (98.2 %)
What form of recording should be used during discharge encounters?	
Audio recording	18 (31.6 %)
Either video or audio	12 (21.1 %)
None	25 (43.9 %)
Video recording	1 (1.8 %)
Missing Values	1 (1.8 %)

**Table 2:** Comfort levels by facility

Characteristic	Non-Academic	Academic	Multiple	Kruskal-Wallis Test P-value
What is your comfort level with having discharge conversations with patients video recorded during an emergency department encounter?				
N	5	27	18	0.76
Mean±SD	49.40 ± 33.74	45.00 ± 38.65	40.33 ± 30.04	
Range	5.00 to 88.00	0.00 to 98.00	0.00 to 87.00	
Median	60.00	34.00	50.00	
What is your comfort level with having discharge conversations with patients audio recorded during an emergency department encounter?				
N	5	23	18	0.16
Mean±SD	51.40 ± 30.75	59.91 ± 38.78	43.83 ± 26.95	
Range	16.00 to 86.00	0.00 to 100.00	0.00 to 87.00	
Median	64.00	82.00	50.00	

Only 20 of the 57 participants surveyed (35%) agreed that patients had the right to record their medical conversations. A total of 36 of the 57 providers (63%) believed that the legal liability of recording outweighed the benefits it could provide to the patient. Twenty-five providers (43.9%) answered that no video or audio recordings should be offered at discharge. Using a Kruskal-Wallis one-way ANOVA statistical variance test, there was no statistically significant difference in response to being recorded for discharge instructions or during any part of the patient encounter. This finding was consistent even when the data were analyzed, looking specifically at the demographics of age, facility type, and sex (Age: p-value 0.57 and 0.22, Facility Type: p-value 0.76 and 0.16, Sex: p-value 0.59 and 0.83). See Tables 2, 3, 4 for this information.

**Table 3:** Comfort levels by age

Characteristic	18-25	26-35	36-50	51-70	Kruskal-Wallis Test P-value
What is your comfort level with having discharge conversations with patients video recorded during an emergency department encounter?					
N	2	21	20	7	0.57
Mean±SD	17.00 ± 24.04	43.81 ± 35.49	42.20 ± 36.06	55.71 ± 32.09	
Range	0.00 to 34.00	0.00 to 96.00	0.00 to 93.00	0.00 to 98.00	
Median	17.00	50.00	32.00	50.00	
What is your comfort level with having discharge conversations with patients audio recorded during an emergency department encounter?					
N	2	19	18	7	0.22
Mean±SD	85.50 ± 20.51	48.32 ± 34.30	47.72 ± 36.44	68.00 ± 22.59	
Range	71.00 to 100.00	0.00 to 96.00	0.00 to 93.00	50.00 to 97.00	
Median	85.50	64.00	50.00	50.00	

**Table 4:** Comfort levels by gender

Characteristic	Female	Male	Wilcoxon Rank Sum Test P-value
What is your comfort level with having discharge conversations with patients video recorded during an emergency department encounter?			
N	25	25	0.59
Mean±SD	35.84 ± 32.95	51.68 ± 35.33	
Range	0.00 to 96.00	0.00 to 98.00	
Median	27.00	58.00	
What is your comfort level with having discharge conversations with patients audio recorded during an emergency department encounter?			
N	22	24	0.83
Mean±SD	50.09 ± 35.30	55.08 ± 33.22	
Range	0.00 to 100.00	0.00 to 97.00	
Median	59.00	57.00	

## Discussion

The primary limitation of this study revolves around the sample size of our study population. Despite our goal of 75 surveys, only 57 were obtained due to our study population's lack of voluntary participation. This diminished sample size likely affected our ability to generate significant power and establish statistical differences between the patient demographics. The lack of power also increases the risk for Type II error and rejects the null hypothesis. This study is also limited to a single metropolitan area and may not apply to all populations secondary to geography and practice patterns. Secondarily to the sample size, it would be difficult to ascertain any significant differences between years of experience or specific provider types. 

The primary goal of this study was to assess emergency medicine providers’ perspectives on being recorded during patient encounters, specifically during discharge conversations. Although the study was underpowered and via a small local sample size, the findings show that there is at least some hesitation among many ED providers to be recorded due to perceived medical-legal concerns. The literature on this topic is mixed. Some studies have demonstrated a potential reduction of medicolegal defense costs in cases where clinicians participated in voluntary recording clinic visits [13]. Conversely, based on this survey, there was no obvious consensus of concerns about recordings adding too much time to the encounters, despite that being cited in other medical literature [4,8].

Currently, there is limited literature on estimates of how often ED patient encounters are being recorded. However, in a mixed-methods analysis of 168 patients, Elwyn et al. demonstrated that approximately 15% of patients in their population admitted to secretly recording during clinical encounters [15]. Although this is not specific to the ED, one can correlate that this is also likely happening in emergency departments. This fact certainly raises concerns about the legality of recording processes. As suspected with the initial hypothesis, many providers cited concern for recordings secondary to medicolegal reasons or possible HIPPA violations.

The HIPPA concerns may be valid as the law is often inconsistently applied in regards to the legality of recording and wiretapping. For instance, 39 of 50 states only require single-party consent for recording encounters, whereas the other 11 require all parties to consent prior [10,16]. This can also vary between video or audio recordings at the state level [16]. Therefore, it may be reasonable for providers to express their concern regarding these, particularly if they are not familiar with local laws surrounding the process. Regarding potential HIPPA violations, if a recording is initiated by the patient and not by the healthcare system, it is not subjective to HIPPA regulations regarding their own encounter [16]. There may be some reasonable concern however within the ED, as it is typically a less controlled environment, and the risk for any recordings involving other patient encounters within the ED may be higher. 

In many of today’s Emergency Departments, both verbal and written discharge instructions are often used. Within the local hospital system surveyed in this study, these instructions are often accompanied by standardized written or prerecorded video instructions based on the diagnosis or chief complaint from that encounter. However, with current technology advancements and readily available cell phones with easy recording capabilities, this topic is continuously gaining interest across emergency medicine literature. As mentioned previously, patients have stated their beliefs in the benefits of recording medical encounters. Physicians are now also expressing potential benefits [17]. This study attempts to determine the perspectives of those working in the Emergency Department and better understand their possible hesitation in being recorded. The study's results show that only 35% of those surveyed felt that patients had the right to record, while 63% felt that the legal liability of recording outweighed the benefits it could provide to the patient. Based on this study, we certainly do not have enough data to change major practices at this time. More literature is needed to achieve any definitive consensus before moving forward and making this a mainstay practice.

## Conclusions

This study highlights that despite the accessibility of patients to the technology to record discharge discussions, health care providers in the ED may still be hesitant about their use of it. We believe further work should be done to see if these findings can be found in other ED provider populations and further tease out why ED providers are hesitant about this practice. 

## References

[1] Butt HR (1977). A method for better physician-patient communication. Ann Intern Med.

[2] Ellis DG, Lerner EB, Jehle DVK, Romano K, Siffring C (1999). A multi-state survey of videotaping practices for major trauma resuscitations. J Emerg Med.

[3] Taylor K, Mayell A, Vandenberg S, Blanchard N, Parshuram CS (2011). Prevalence and indications for video recording in the health care setting in North American and British paediatric hospitals. Paediatr Child Health.

[4] McConnell D, Butow PN, Tattersall MH (1999). Audiotapes and letters to patients: the practice and views of oncologists, surgeons and general practitioners. Br J Cancer.

[5] Meeusen AJ, Porter R (2015). Patient-reported use of personalized video recordings to improve neurosurgical patient-provider communication. Cureus.

[6] Campbell S, Sosa JA, Rabinovici R, Frankel H (2006). Do not roll the videotape: effects of the health insurance portability and accountability act and the law on trauma videotaping practices. Am J Surg.

[7] Tattersall MH, Butow PN, Griffin AM, Dunn SM (1994). The take-home message: patients prefer consultation audiotapes to summary letters. J Clin Oncol.

[8] Belkora JK, Loth MK, Chen DF, Chen JY, Volz S, Esserman LJ (2008). Monitoring the implementation of consultation planning, recording, and summarizing in a breast care center. Patient Educ Couns.

[9] Liddell C, Rae G, Brown TRM, Johnston D, Coates V, Mallett J (2004). Giving patients an audiotape of their GP consultation: a randomised controlled trial. Br J Gen Pract.

[10] Dolan PL (2012). Pros and cons of letting patients record doctor visits. Am Med News.

[11] Rodriguez M, Morrow J, Seifi A (2015). Ethical implications of patients and families secretly recording conversations with physicians. JAMA.

[12] Henken KR, Jansen FW, Klein J, Stassen LP, Dankelman J, van den Dobbelsteen JJ (2012). Implications of the law on video recording in clinical practice. Surg Endosc.

[13] (2019). Health Privacy: HIPAA Basics | Privacy Rights Clearinghouse. https://www.privacyrights.org/consumer-guides/health-privacy-hipaa-basics.

[14] Audiovisual Recording in the Emergency Department (2019). ACEP // Audiovisual Recording in the Emergency Department. American College of Emergency Physicians. June.

[15] Elwyn G, Barr PJ, Grande SW (2015). Patients recording clinical encounters: a path to empowerment? Assessment by mixed methods. BMJ Open.

[16] Elwyn G, Barr PJ, Castaldo M (2017). Can patients make recordings of medical encounters? What does the law say?. JAMA.

[17] Ballard Ballard, Dustin MD (2021). Medically clear. Even the ED may have room for recordings. Emerg Med News.

